# Sir H. Thompson on Urinary Organs

**Published:** 1874-12

**Authors:** 


Clinical Lectures on Diseases of the Urinary Organs. Delivered at University College Hospital. By Sir Henry Thompson, Surgeon Extraordinary to His Majesty the King of the Belgians; Professor of Clinical Surgery, and Surgeon to University College Hospital. Second American, from the third and revised English edition; with illustrations. Philadelphia: Henry C. Lea, 1874. Octavo; cloth; pp. 195. Price $2.25.

This work, which embodies the course of clinical lectures upon the urinary organs, delivered in University College Hospital during the winter of 1872–3, by so ripe a scholar and so distinguished a specialist as Sir Henry Thompson is everywhere considered to be, is above and beyond our criticism. To those of our readers who are interested in this specialty, we need only announce the appearance of the work.

